# Hospital Finance and Economy

**Published:** 1888-08-25

**Authors:** 


### HOSPITAL FINANCE AND ECONOMY.

BY A HOSPITAL SUPERINTENDENT.

HAS any one yet been able to formulate a hospital balance-sheet acceptable to all, one which could determine the cost of each patient treated, and be used as a standard for comparison with other institutions? We are forced to ask this question after wading through a miscellaneous collection of hospital reports, and having been fairly baffled in our attempt to analyse them with the view of arriving at a satisfactory estimate of the cost of an occupied bed in each establishment. No two of these documents are conceived on the same lines; all are more or less tainted with a spirit of self-assumption, which is the bane of local self-government, and they in a measure defies analysis and comparison. How those quotations one frequently meets with in the daily and weekly press, purporting to give the cost of a bed in this or other hospital pass current as authentic data is a mystery to every one versed in hospital finance. Someone must have fathered them in the first instance, but when we come to inquire into their veracity, we may be certain that exception will be taken to the figures. It was consequently with a feeling of relief we received a copy of the statistics prepared for the Council of the Hospital Sunday Fund, which are drawn up on a uniform principle, and cannot be accused of bias in any direction. Here, if anywhere, we ought to be able to discover the needs and merits of London hospitals, at all events; and after a careful study of the figures, notwithstanding some shortcomings in their application, we have not been disappointed. In making their awards the committee of distribution have required the representatives of 109 hospitals and 50 dispensaries to furnish them with detailed information respecting the cost per week of each occupied bed, the expense of each out-patient, the mean income and expenditure of each establishment for the last three years, and other facts bearing on its constitution and work. These data are tabulated in columns, and present to the curious in such matters a wealth of information, which, albeit somewhat perplexing to the uninitiated, is extremely interesting and instructive. In their desire to be judicial, the committee have inserted a column in their tables differentiating between the cost of management and maintenance—no doubt with the intention of favouring such establishments as are economically administered; but it is difficult to draw a hard and fast line between the departments, which must be left in a measure to the discretion of the party contributing the information. The fact of the percentage cost of management to maintenance ranging in the returns made from 1 to 45 per cent. is significant of the difficulties surrounding the question. But a greater and more suggestive discrepancy awaits us when we come to analyse the cost of an occupied bed as stated by hospital authorities. To reduce the notoriously high average cost of in-patients in London hospitals, the Council of the Fund have very generously added a column to their tables, by which the anomalous expenditure may be thrown to a large extent on the out-patients. Now, everyone acquainted with hospitals must be fully aware of the great fallacies which underlie the enumeration of out-patients, and the impracticability of separating the expenses of this department from the other. Scarcely two hospitals adopt the same principles of classification and computation. It will emphasise this fact if we draw our conclusions from the figures returned to the Council by the hospital authorities themselves, which show that the cost of an out-patient at the different hospitals oscillates on a scale ranging from one shilling a head to thirty-eight shillings and ninepence, without any indication that the patient was better treated in the latter establishment than in the former. It seems also pretty evident from the statistics that the institution which spends so much money on its out-patients is also extravagant in the cost of its in-patients. The only standard admitting of uniformity and comparison must be arrived at by dividing the annual expenditure by the mean number of patients occupying beds during the year, omitting the expense of new buildings, which are usually provided for by a separate fund. This is the plan adopted by foreign countries, by our own Local Government Board with reference to infirmaries and sick asylums, by provincial hospitals, and, in fact, by all establishments except in London.

By pursuing the line of inquiry in the manner indicated, with the figures supplied for the Council of the Hospital Sunday Fund, we will attempt to arrive at the annual cost of a patient in the institutions referred to. Perhaps it would be better to take the hospitals associated with medical schools first, as, on account of their clinical advantages, they are generally assumed to be more expensively conducted than other and special hospitals. This is not always shown to be the case, although it is pretty frequently so. We have added a column to the tables, showing the awards paid over to the respective institutions by the Distribution Committee of the Hospital Fund, on a principle very open to objection, but which we refrain from commenting on at present.

The cost per head has been computed from the gross average expenditure of each institution during the last three years, excluding investments of legacies, repayment of mortgages, cost of new buildings, etc., and is divided by the mean number of patients resident in the hospital during the past year. It would no doubt have been better to have had the average number of resident patients for the three years instead of for one year only :—

| Name of Hospital. | Average No. of Occupied Beds. | Cost per Head per Annum. | Award Paid Over 1888. |
|---|---|---|---|
| Charing Cross Hospital | 125 | £108 | £989 |
| Guy's Hospital | 410 | 81 | 462 |
| King's College Hospital | 151 | 130 | 1,354 |
| The London Hospital | 644 | 84 | 3,317 |
| Royal Free Hospital | 135 | 83 | 958 |
| St. George's Hospital | 277 | 92 | 1,562 |
| St. Mary's Hospital | 243 | 79 | 1,769 |
| The Middlesex Hospital | 176 | 143 | 2,031 |
| University College Hospital | 179 | 116 | 1,351 |
| Westminster Hospital | 172 | 89 | 1,041 |

This gives a mean cost of £100 a bed at ten of the clinical hospitals, and there is no reason to suppose that in the remaining two—St. Bartholomew's and St. Thomas's—the patients can be kept at a less cost. We will now take the smaller general hospitals and treat them in a similar way to the scholastic establishments.

| Name of Hospital. | Average No. of Occupied Beds. | Annual Cost per Head. | Award Paid Over, 1888. |
|---|---|---|---|
| French Hospital | 28 | £96 | £208 |
| German Hospital | 110 | 84 | 662 |
| Great Northern Central | 29 | 123 | 227 |
| Italian Hospital | 12 | 63 | 73 |
| Miller Hospital, Greenwich | 19 | 176 | 285 |
| North-West London Hospital | 36 | 92 | 291 |
| Poplar Hospital | 35 | 89 | 218 |
| Seamen's Hospital | 190 | 60 | 625 |
| Training Hospital, Tottenham | 52 | 58 | 260 |
| West London Hospital | 81 | 63 | 520 |

Although the smaller general hospitals are conducted at a less average cost (£90) than the larger establishments, it will be noticed that they vary more with regard to their individual cost. This fact becomes more recognized and accentuated when we come to consider the maintenance and management of the host of special hospitals which have sprung into existence during the last half-century, and the number of which is continually being added to. We will first refer to the cost of the children's hospitals, which present features calling for special inquiry on the part of their supporters :—

| | Occupied Beds. | Annual Cost per Head. | Award Paid Over. 1888. |
|---|---|---|---|
| Alexandra Hospital for Hip Disease in Childhood | 80 | £30 | £177 |
| Belgrave Hospital for Children | 13 | 103 | 135 |
| Shadwell Hospital for Children | 74 | 87 | 416 |
| Evelina Hospital, Southwark | 55 | 91 | 458 |
| Great Ormond Street Hospital | 126 | 77 | 729 |
| North-Eastern, Hackney Road | 50 | 96 | 333 |
| Paddington Green Hospital | 20 | 102 | 163 |
| Victoria Hospital, Chelsea | 66 | 72 | 333 |
| The Vine, Sevenoaks | 17 | 56 | 20 |

Some of the miscelleneous special hospitals present equally remarkable features which ought to elicit public animadversion :—

| Name of Hospital. | Occupied Beds. | Annual Cost per Head. | Award Paid Over, 1888. |
|---|---|---|---|
| Victoria Pk. Hosp. for Chest Disease | 107 | £95 | £866 |
| Brompton Hosp. for Consumption | 232 | 102 | 1,872 |
| N. London Hosp. for Consumption | 37 | 120 | 208 |
| Hosp. for Dis. of Chest, City Road | 36 | 107 | 312 |
| Consumption Hospital, Ventnor | 115 | 82 | 208 |
| Chelsea Hospital for Women | 45 | 71 | 192 |
| Hospital for Women, Soho Square | 53 | 115 | 427 |
| Grosvenor Hospital, Women and Children | 11 | 88 | 83 |
| New Hospital, Women, Marylebone Road | 20 | 98 | 125 |
| Royal Hospital, Women and Children, Lambeth | 47 | 69 | 218 |
| Samaritan Hosp., Seymour Street | 48 | 108 | 479 |
| Cancer Hospital, Brompton | 77 | 106 | 468 |
| St. Saviour's Cancer Hospital | 25 | 127 | — |
| London Fever, Liverpool Road | 100 | 85 | 572 |
| St. Mark's Hospital for Fistula | 24 | 91 | 93 |
| Hosp. for Heart Disease, Soho Sq. | 20 | £104 | £140 |
| Female Lock Hospital | 66 | 64 | 260 |
| Male Lock Hospital | 14 | 93 | 72 |
| Hospital for Epilepsy and Paralysis | 17 | 118 | 83 |
| National Hospital for Paralysis and Epilepsy | 110 | 69 | 416 |
| Royal London Ophthalmic | 93 | 67 | 572 |
| Royal Westminster Ophthalmic | 22 | 78 | 88 |
| City Orthopædic Hospital | 15 | 93 | 78 |
| National Orthopædic Hospital | 34 | 32 | 31 |
| Royal Orthopædic Hospital | 47 | 43 | 88 |
| Throat and Ear, Central London Hospital | 14 | 127 | 57 |
| Hospital for Throat Diseases | 15 | 180 | 36 |
| London Homœopathic Hospital | 53 | 102 | 208 |
| London Temperance Hospital | 50 | 83 | 360 |

In our analysis we have adhered to hospitals only, and have purposely reserved lying-in establishments, convalescent homes, and dispensaries for another occasion. The figures prove beyond all doubt which institutions are most deserving of support if economy of management is to be considered an element of efficiency; but we fail in very many instances to discover the cause of such a marked disproportion in the character of the awards. At the same time we are ready to admit the unwisdom of forming hasty conclusions from the figures as they stand. It may be contended that in many instances the lavish expenditure per patient has been caused by the closing of the hospital for a part of the year—an arbitrary proceeding, throwing doubts on its *raison d'etre*, but one which enormously increases the average cost, by virtue of the numerical reduction.

